# The policy of free healthcare for children under the age of 6 years in Vietnam: assessment of the uptake for children hospitalised with acute diarrhoea in Ho Chi Minh City

**DOI:** 10.1111/tmi.12208

**Published:** 2013-10-18

**Authors:** Mae Shieh, Corinne Thompson, My Phan Vu Tra, Van Thi Thuy Linh, Fabrizio Tediosi, Laura Merson, Jeremy J Farrar, Ha Manh Tuan, Ho Lu Viet, Pham Thi Ngoc Tuyet, Stephen Baker

**Affiliations:** 1 Novartis Vaccines Institute for Global HealthSiena, Italy; 2 Hospital for Tropical Diseases, Wellcome Trust Major Overseas Programme, Oxford University Clinical Research UnitHo Chi Minh City, Vietnam; 3 Nuffield Department of Clinical Medicine, Centre for Tropical Medicine, Oxford UniversityOxford, UK; 4 Children's Hospital 2Ho Chi Minh City, Vietnam; 5 Swiss Tropical and Public Health InstituteBasel, Switzerland; 6 London School of Hygiene and Tropical MedicineLondon, UK

**Keywords:** social health insurance, health insurance utilisation, child health, acute diarrhoea, out-of-pocket expenditures, hospital charges, Vietnam

## Abstract

**Objective:**

To assess the proportion of, and reasons for, households not utilising the policy of free healthcare for children under 6 years of age (FCCU6) for hospitalisation with diarrhoea, and assess the risk of catastrophic expenditure for households that forgo FCCU6 and pay out of pocket.

**Methods:**

Invoices detailing insurance information and charges incurred from 472 hospitalised diarrhoeal cases in one paediatric hospital in Ho Chi Minh City were retrieved. Hospital charges and the utilisation of elective services were analysed for patients utilising and not utilising FCCU6. Associations between socio-economic factors with non-utilisation of FCCU6 were evaluated.

**Results:**

Overall, 29% of patients were FCCU6 non-users. The FCCU6 non-users paid a median hospital charge of $29.13 (interquartile range, IQR: $18.57–46.24), consuming no more than 1.4% of a medium-income household's annual income. Seventy per cent of low-income FCCU6 non-users utilised less-expensive elective services, whereas only 43% of medium income patients and 21% of high-income patients did (*P* = 0.036). Patients from larger households and those with a parent working in government were more likely to use FCCU6.

**Conclusions:**

The rate of FCCU6 non-usage in this study population was 29%. A significant proportion of those that did not use FCCU6 was from lower income households and may perceive a justifiable cost–benefit ratio when forgoing FCCU6. Although a single diarrhoeal hospitalisation is unlikely to induce a catastrophic expenditure, FCCU6 non-usage may disproportionately increase the risk of catastrophic expenditure for lower income households over multiple illnesses.

## Introduction

Vietnam has one of the highest percentages of households at-risk of catastrophic health expenditures (expenses potentially driving households into poverty) among East Asian countries in a comparable stage of development (Xu *et al*. 2003; van Doorslaer *et al*. 2006, 2007). The Vietnamese government has aimed to reduce out-of-pocket (OOP) health expenditures for households through the introduction of universal health insurance, which includes special policies providing free healthcare to the poor and children under 6 years old (FCCU6). The state contributes the premiums for both groups, who are then protected under the compulsory social health insurance scheme. The voluntary scheme covers the rest of the population, where premiums are paid on a voluntary basis. FCCU6 was implemented nationwide in 2005. Under this policy children under the age of 6 years old are entitled to no-cost treatment, laboratory tests and generic medications at public outpatient and inpatient facilities. In order to access this free healthcare, the child must hold a government health insurance card and access healthcare at the local public facility to which they are registered. Tertiary facilities are only accessible with a formal referral. In 2010, it was reported that 8.2 million of the 10.1 million (81%) children under 6 years of age in Vietnam had health insurance cards (MOH/HPG 2010). Parents/guardians in possession of a health insurance card for their child may still choose to forgo FCCU6 and pay OOP should they wish to bypass standard facilities and take their children directly to tertiary referral hospitals, often regarded as offering higher quality services (Trivedi 2002). Nguyen and Wang assessed healthcare facility usage of children less than 6 years of age in the period prior to, and directly after, FCCU6 was implemented nationwide (Nguyen & Wang 2012). This study found a decrease in the use of tertiary facilities and an increase in use of secondary healthcare facilities, which was perceived to be a desirable consequence of formal referral being required for payment by insurance under FCCU6. The percentage of households utilising FCCU6 was not assessed.

Vietnam, as in other Southeast Asian countries, has undergone reforms in its health-financing system, moving from a completely state-led and financed system to one where public hospitals have autonomy in their administration and budgets (Tangcharoensathien *et al*. 2011). It has been reported that in response to the marked increase in number of children presenting at already over-crowded paediatric hospitals after the implementation of FCCU6, hospitals in Hanoi and Ho Chi Minh City began to offer a ‘non-policy’ option, where patients who chose not to use FCCU6 received preferential treatment, for example, a shorter waiting time (London 2008). Furthermore, public hospitals are also permitted to offer patients non-essential elective services, such as special care in private wards. If the patient chooses to use any non-essential elective service they cannot use state-provided health insurance and must pay OOP for the entire hospital admission, as hospitals are required to keep private and public services separate (World Bank Vietnam Office 2011). Although the overall proportion of the population with health insurance in Vietnam has been increasing, the use of health insurance actually declined between 2006 and 2008 and OOP payments remain high, estimated to be >50% of all the healthcare financing in Vietnam (MOH/HPG 2010).

Childhood health is a high priority for Vietnam but little has been reported on the usage and factors affecting usage of FCCU6, which was put in place to specifically protect this vulnerable group. By assessing hospital charges, predictive factors, and the risk of catastrophic expenditure we aimed to understand how FCCU6 is performing in children hospitalised with acute diarrhoea in a tertiary hospital in Ho Chi Minh City (HCMC).

## Methods

### Study design

This study was performed retrospectively on available data from patients that were enrolled in a prospective observational study conducted between May 2009 and April 2010 in HCMC (Thompson *et al*. 2012).

### Study site and subjects

The observational study, from which the subjects of this analysis were derived, was conducted by Oxford University Clinical Research Unit (OUCRU) at Children's Hospital 2 in HCMC. Children's Hospital 2 is one of the main paediatric referral hospitals in HCMC. Children <5 years old, residing in HCMC, admitted to Children's Hospital 2 with acute diarrhoea were eligible for the observational study. For this retrospective analysis, further inclusion criteria were the availability of the final hospital invoice, and an unambiguous payment category (i.e. paid entirely by social insurance under FCCU6 or OOP by household). Demographic data were collected on hospital admission and categorised where appropriate. For example, household income was stratified in to three income categories (low; <173 USD/month, medium; 173–865 USD/month; >865 USD/month) based on average household incomes (in Vietnamese Dong) and demographics from a previous investigation (Thompson *et al*. 2012).

### Data source

Two individuals independently entered information from the hospital invoice, including payment type, type of service, quantities and the unit costs of medications, medical tests, services and supplies. These data (from each patient) were collated into a customised database, and were combined with demographic, socioeconomic and clinical information from the observational study.

### Definitions and unit costs of services

The total charge for each patient's hospitalisation was calculated as the sum of the charges incurred for medication, laboratory tests, hospital bed overnight charges, uncommon services and elective services incurred until discharge, using the definitions as described below. A standard hospital bed in the gastrointestinal ward, with two or more children per bed, had an associated overnight charge of $0.58–1.04 USD/day. A special service bed, with one child per bed, air conditioning and television, was considered a non-essential elective service, unless medically warranted due to the severity of the patient's condition, and was available for $5.77 and up to $17.00/day (referred to hereon as ‘private bed’). Patients in this study with an average daily bed charge of more than $1.04 were presumed to have stayed at least one night in a private bed. In addition to private beds, other elective services included bed cleaning, charged at $0.29 each, and meals (e.g. porridge, rice) available for $0.86–2.00 each (referred to collectively hereon as cleaning/meal services, whenever either or both was utilised). Uncommon services for diarrhoeal patients included ultrasound, X-ray and physical therapy, charged at $3.46, $6.90 and $0.86 per unit, respectively. Uncommon services were not considered elective. All charges were measured in local currency in 2009 Vietnamese Dong (VND) and converted to 2009 USD using the average June 2009 exchange rate of 17 339 VND/USD.

### Data analysis

Utilisation of elective services was analysed by household income category and payment type. Logistic regression was performed to examine the variables associated with households who paid OOP. Potential determinants for paying OOP (sex, age, parent job type, household size, income category and distance to hospital) were assessed independently by univariate analysis. Variables strongly associated with the outcome in the univariate analysis (*P* ≤ 0.05), in addition to *a-priori* variables considered potential confounders, were included in a multivariate logistic regression model to evaluate independent associations with paying OOP.

All statistical analyses were conducted using Stata/IC version 9.2 (StataCorp, USA). A two-tailed Student's *t*-test or a corresponding non-parametric test (Mann–Whitney *U*, Kruskal–Wallis) was performed on numerical data for paired and group comparisons respectively. The Chi-squared test was used to compare proportions between categorical variables. A *P*-value ≤ 0.05 was considered statistically significant.

### Ethical approval

This study was conducted according to the principles expressed in the Declaration of Helsinki. This work underwent ethical review and was approved by the institutional ethical review board of Children's Hospital 2 and the Oxford Tropical Research Ethics Committee (OXTREC) in the United Kingdom. The parents/guardians of the children were required to provide written informed consent for the collection of samples and subsequent analysis.

## Results

### Payment type

**Figure 1:**
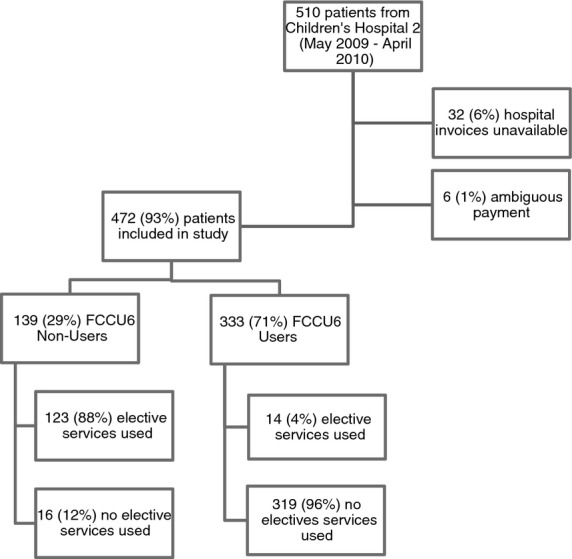
Flowchart of the patients included in the study and breakdown by payment type and elective service use.

A flowchart of the study population is shown in Figure 1. Of 510 diarrhoeal cases enrolled in an observational study conducted at Children's Hospital 2 between May 2009 and April 2010, final hospital invoices were available from 478 (94%) admissions. For six (1.2%) patients, the household paid partially OOP, with insurance paying the remaining balance. These mixed payment types were exceptional and were excluded from all subsequent analyses. For the remaining 472 (93%) patients, hospital expenses were paid either entirely via the social insurance system (FCCU6 users) or entirely OOP by the household (FCCU6 non-users). In this study population, 333/472 (71%) of the patients were FCCU6 users, while 139/472 (29%) were FCCU6 non-users. Further, 88% (123/139) of FCCU6 non-users utilised at least one elective service, compared to 4% (14/333) of FCCU6 users (*P *<* *0.001; Chi-squared test).

### Patient characteristics

The baseline characteristics of the 472 diarrhoeal patients overall and stratified by payment type are shown in Table 1. The children admitted to Children's Hospital 2 with diarrhoeal disease contributing to this study had a median age of 14 months (interquartile range, IQR: 9–21 months), were more commonly male (302/472; 64%), and less frequently from high-income households (55/472; 12%). We found that a higher proportion of parents of the FCCU6 users had a government job (42/333, 13%) compared to FCCU6 non-users (9/139, 6%; *P *=* *0.05; Chi-squared test). Furthermore, FCCU6 users had more family members than non-users [median: 6 (IQR: 5–8) and 5 (IQR: 4–7), respectively, *P *<* *0.001, Mann–Whitney *U*-test]. After controlling for the confounding effect of age, sex and income level in a multivariate logistic regression model, the variables significantly associated with payment type were household size and having a parent with a government job. Larger households (OR = 0.87, 95% CI: 0.81–0.96) and those with a parent working in the government (OR = 0.41, 95% CI: 0.19–0.89) were significantly less likely to pay OOP (Table 2).

**Table 1:** Baseline characteristics of all patients stratified by payment type

Variable	All	FCCU6	Out-of-pocket
No. (%)	472 (100)	333 (71)	139 (29)
Male gender – No. (%)	302 (64)	212 (64)	90 (65)
Age in months – Median (interquartile range, IQR)	14 (9–21)	13 (8–21)	14 (9–20)
Household size – Median (IQR)	6 (3–8)	6 (5–8)*	5 (4–7)*
Household income category† – No. (%)			
Low (<$173/month)	116 (24)	86 (26)	30 (21)
Medium ($173–865/month)	301 (64)	211 (63)	90 (65)
High (>$865/month)	55 (12)	36 (11)	19 (14)
Distance to Children's Hospital 2 (km) – Median (IQR)	7.1 (3.6–10.4)	7.0 (3.3–9.9)	7.3 (4.1–10.8)
Parent with government job – No (%)	51 (11)	42 (13)*	9 (6)*

* *P *<* *0.001.

† Income thresholds in 2009 USD, using 2009 exchange rate of 17 339 Vietnamese Dong/USD.

**Table 2:** Univariate and multivariate analysis of selected out-of-pocket determinants

Variable	OR	95% CI	*P*	aOR	95% CI	*P*
Male gender	1.05	0.69–1.59	0.823	1.1	0.72–1.68	0.661
Age in months	0.99	0.98–1.02	0.743	0.99	0.97–1.01	0.588
Income category						
Low (<$173/month)	1.00	–	–	1.00	–	–
Medium ($173–865/month)	1.22	0.75–1.98	0.415	1.35	0.83–2.21	0.229
High (>$865/month)	1.51	0.76–3.03	0.242	1.94	0.94–3.99	0.072
Household size	0.89	0.81–0.97	0.006	0.88	0.81–0.96	0.004
Parent with government job	0.48	0.23–1.01	0.055	0.41	0.19–0.89	0.024

OR, odds ratio; CI, confidence interval; aOR, adjusted odds ratio.

### Comparison between FCCU6 users and non-users

The healthcare utilisation of the 472 patients stratified by payment type is shown in Table 3. The median total charge for the entire period of hospitalisation for an episode of acute diarrhoea for FCCU6 non-users was 1.6 times greater [$29.13 (IQR: $18.57–46.24)] than FCCU6 users [$18.50 (IQR: $12.96–26.86)] (*P *<* *0.001; Mann–Whitney *U*-test). FCCU6 users had a higher median charge for medication ($5.79 (IQR: $3.95–7.99) than FCCU6 non-users ($4.02 (IQR: $2.82–6.86), while FCCU6 non-users had a higher median bed charge ($4.33 (IQR: $2.02–23.88) than FCCU6 users ($2.30 (IQR: $1.15–3.46). FCCU6 non-users paid more than FCCU6 users in the high (*P *=* *0.003) and medium (*P *<* *0.001) income groups, but not in the low-income group (*P *=* *0.341; Mann–Whitney *U*-test). 15% (70/472) of all patients utilised a private bed for at least one night during the hospital stay, incurring a 9.7 times greater median per-day bed charge than those staying in a standard bed ($4.74 *vs*. $0.49). Twenty-two per cent (106/472) of all patients utilised cleaning services and/or meals, incurring a median charge of $2.31 (IQR: $1.15–4.04). More FCCU6 non-users stayed in private beds (59/139, 42% *vs*. 11/333, 3%; Chi-squared test, *P *<* *0.001) and utilised a cleaning and/or meal service (100/139, 72% *vs*. 6/333, 1.8%; Chi-squared test, *P *<* *0.001) than FCCU6 users. As the households of FCCU6 non-users paid OOP, the hospital charges consumed <0.4% of a high-income household's annual income, 0.16–1.4% of a medium-income household's annual income, and >1.2% of a low-income household's income.

**Table 3:** Healthcare utilisation of all patients and stratified by payment type

Variable	All	FCCU6	Out-of-pocket
No. (%)	472 (100)	333 (71)	139 (29)
Total charge ($†) – Median (interquartile range, IQR)	20.31 (14.11–32.33)	18.50 (12.96–26.86)*	29.13 (18.57–46.24)*
Medication charges ($) – Median (IQR)	5.36 (3.61–7.81)	5.79 (3.95–7.99)*	4.02 (2.82–6.86)*
Laboratory test charges ($) – Median (IQR)	8.31 (3.46–19.90)	8.02 (3.11–10.32)	8.31 (3.98–11.13)
Bed charges ($) – Median (IQR)	2.88 (1.53–4.61)	2.30 (1.15–3.46)*	4.33 (2.02–23.88)*
Total charge by household income category ($) – Median (IQR)			
Low (<$173/month)	20.32 (14.45–30.04)	19.67 (14.91–$31.10)	24.47 (13.22–40.61)
Medium ($173–865/month)	19.47 (13.51–29.90)	16.65 (12.55–25.27)*	29.25 (18.57–41.96)*
High (>$865/month)	24.91 (18.72–49.57)	22.21 (16.26–35.41)*	40.10 (24.91–57.35)*
Length of stay in hospital (days) – Median (IQR)	5 (2–7)	5 (4–7)*	5 (3–6)*
Uncommon services – No. (%)	87 (18)	65 (19)	22 (16)
Private bed – No. (%)	70 (15)	11 (3)*	59 (42)*
Cleaning or meal service – No. (%)	106 (22)	6 (1.8)*	100 (72)*

* *P *<* *0.01.

† Charges in 2009 USD, using 2009 exchange rate of 17 339 Vietnamese Dong/USD.

### Comparison of elective service utilisation by income category

There was no significant disparity between the proportion of low, medium and high-income households (30/116, 26%; 90/301, 30%; and 19/55; 35%, respectively; *P *=* *0.603; Chi-squared test) that did not use FCCU6. The breakdown of the elective services for FCCU6 users and non-users by income category is shown in Table 4. Among the FCCU6 non-users, low-income households (21/30, 70%) utilised elective cleaning and/or meal services (but not a private bed) more commonly than medium (39/90, 43%) and high-income households (4/19, 21%; *P *=* *0.036; Chi-squared test). FCCU6 non-users from high-income households (15/19, 79%) were more likely to use a private bed than FCCU6 non-users from medium (40/90, 44%) and low-income (4/30, 13%) households (*P *<* *0.01; Chi-squared test). Among FCCU6 non-users, more low-income (5/30, 17%) than medium (11/90, 12%) and high-income (0/19, 0%) households did not use any elective services, but this did not reach statistical significance (*P *=* *0.232; Chi-squared test). FCCU6 users had similar usage of elective services across all income categories.

**Table 4:** Utilisation of elective services by payment type and income category

		Household income category†		
Variable	All	Low	Medium	High
No. (%)	472 (100)	116 (24)	301 (64)	55 (12)
Out-of-pocket by household – No. (%)	139 (29)	30 (21)	90 (65)	19 (14)
Use of private bed – No. (%)	59 (42)	4 (13)*	40 (45)*	15 (79)*
Use of cleaning/meal only – No. (%)	64 (46)	21 (70)*	39 (43)*	4 (21)*
No elective services used – No. (%)	16 (12)	5 (17)	11 (12)	0 (0)
FCCU6 by insurance – No. (%)	333 (71)	86 (74)	211 (70)	36 (65)
Use of private bed – No. (%)	11 (3)	4 (5)	6 (3)	1 (3)
Use of cleaning/meal only – No. (%)	3 (1)	1 (1)	2 (1)	0 (0)
No elective services used – No. (%)	319 (96)	81 (94)	203 (96)	35 (97)

* *P *<* *0.01.

† Low <$173/month, Medium $173–865/month, High >$865/month.

## Discussion

This study aimed to assess the proportion of households that did not utilise FCCU6 for the hospitalisation of a common childhood disease and add some insight into why some may not use this government healthcare cover. Our findings show that 29% of the study population did not use FCCU6 and paid a median total OOP charge of $29.13, consuming no more than 1.4% of a medium-income household's annual income. Furthermore, we found that the types of elective services used differed by income category among the FCCU6 non-users and the factors associated with FCCU6 uptake included having a larger household and having a parent with a government job.

In 2009, Sepehri *et al*. reported an inpatient insurance usage rate of 81% (data taken from the 2006 Vietnam Household Living Standard Survey) for those over the age of 6 years in Vietnam (Sepehri *et al*. 2009). Our study population was largely drawn from urban households, as most travelled a relatively short distance to reach this hospital in central HCMC, therefore, was potentially biased towards urban households. As urban household have better access to health insurance than rural households (Hoa *et al*. 2007), the 71% FCCU6 utilisation rate observed in our study is substantially lower than would be predicted in this study population. If the intention of FCCU6 is to prevent undue financial burden on vulnerable households, policy makers may want to ensure that more low-income than higher income households use FCCU6. Our results showed a trend towards higher FCCU6 uptake from households with lower incomes (with respect to higher income households), however, this was not statistically significant.

For the FCCU6 non-users, the hospital charges incurred during hospitalisation for diarrhoea were paid OOP, thereby increasing the household's risk of catastrophic spending [defined as 30–40% of a household's capacity to pay, or 10% of annual household expenditure (Wagstaff & van Doorslaer 2003)]. Although we could not directly assess the capacity of individual households to pay, our results showed that a single hospitalisation for diarrhoeal disease consumed less than 10% of a medium and high-income household's annual income and of low-income households earning more than $20/month. Although we cannot ascertain what proportion of low-income households earned less than $20/month, this catastrophic expenditure threshold is 8 times lower than the low-income threshold of $173/month. Therefore, our results suggest that diarrhoeal hospitalisation, as a single event, is not likely to be a catastrophic expenditure in this study population. However, repeated OOP payments increase the risk of catastrophic spending over the course of a year, particularly within low-income households. Thuan reported that >92% (2533/2727) of individuals resident in the northwest of Vietnam had at least one illness a year, with an average of three episodes per year (Thuan *et al*. 2006). Further, the author concluded that catastrophic spending does not usually occur from one catastrophic illness, but rather an accumulation of ‘everyday’ episodes. As low-income households have approximately three times less income than high-income households and only 1.6 times lower median hospital charge, the likelihood of catastrophic medical spending is proportionally higher for low-income households.

Health-seeking behaviour and use of health insurance has been long recognised as complex and multifaceted. Sepehri *et al*. proposed that those covered by a policy providing free healthcare for the poor were less likely to access insurance benefits than those paying premiums under compulsory insurance (Sepehri *et al*. 2009). It was also suggested by Hoa *et al*. that households were motivated to seek higher quality care for children with more severe medical conditions, and that urban households described payment for medical treatment as ‘more acceptable and less expensive’ than rural households (Hoa *et al*. 2007). We found that FCCU6 non-users who utilised a private bed were more frequently from high-income households, while FCCU6 non-users who only utilised less-expensive cleaning and/or meal service were more commonly lower income patients. One potential explanation for the latter finding is that low-income households, who could not afford private beds, could, by utilising the less expensive cleaning and/or meal service, still access perceived or actual higher quality non-policy services and benefits, e.g., a shorter waiting time than FCCU6 users, and/or avoid the extensive paperwork required to use FCCU6 (UNICEF 2011). These findings suggest that some households perceived the severity of the diarrhoea to be sufficiently high and the OOP expenditure for the diarrhoeal hospitalisation to be sufficiently low to motivate the household to forgo FCCU6 and pay OOP. Our results demonstrating a low likelihood of a single hospitalisation to induce a catastrophic expenditure further supports this conclusion. We surmise that the small proportion of FCCU6 non-users who did not use either elective service were forced to forgo FCCU6 and pay OOP due to not fulfilling the FCCU6 requirements, that is, no insurance card or appropriate referral.

The parents/guardians who were motivated to utilise FCCU6 were more likely to come from larger households and to have a government job. A larger family may limit the ability of a parent to pay additional non-food costs, including healthcare, which may result in a greater motivation to use FCCU6. Those with government jobs may be more aware of the procedures for accessing insurance more efficiently. Additionally, previous research has reported inequitable treatment by healthcare staff towards patients of lower social position, for example, women, ethnic minorities (Rheinlander *et al*. 2011; Malqvist *et al*. 2012, 2013). Consequently, patients who have a parent in a respected government positions may be perceived as having a high social position and receive preferential treatment by healthcare staff without forgoing FCCU6. In the usage of free maternal healthcare in Pakistan, those from the richest societal class were the main users of free public services, while middle and poorer women often paid for private services as a sign of higher social status (Mumtaz *et al*. 2013). Further research is needed to determine if this behaviour is present and influences FCCU6 utilisation in Vietnam.

Our research has some limitations, which need to be considered before placing our findings into a greater context. Previous studies have suggested that health-seeking behaviour is different between serious and less serious conditions (Lieberman & Wagstaff 2009; Kaljee *et al*. 2011). Therefore, our results may not be representative when the same households are faced with medical conditions of different severity than acute diarrhoea resulting in hospitalisation. All children enrolled in this study were resident in HCMC and had symptoms for less than 3 days prior to seeking healthcare, suggesting that Children's Hospital 2 was the designated primary healthcare facility for FCCU6. However, we cannot exclude the possibility that the patients who used the cleaning and/or meal services and paid OOP did not have an insurance card or appropriate referral. Consequently, our investigation was not able to definitively determine if these households did not use FCCU6 through choice (for higher quality treatment) or through necessity. It is noteworthy that the hospital selected for this study was a tertiary referral hospital. However, this is unlikely to introduce selection bias as the children admitted to this hospital with diarrhoea are required to present first as outpatients and there is currently no formal policy for referral due to diarrhoea.

## Conclusions

Our results demonstrated that the FCCU6 non-usage rate in this study population in HCMC was 29%. A substantial proportion of those forgoing FCCU6 was from lower income households, and may perceive paying OOP for healthcare to be of a justifiable cost–benefit. Hospitalisation for diarrhoea in this population is unlikely to induce a catastrophic expenditure, yet FCCU6 non-usage may disproportionately increase the risk of catastrophic expenditure for lower income households throughout multiple illness episodes. Measures to improve capacity of and incentives for hospitals to provide FCCU6 users with higher quality services may increase the usage of FCCU6, particularly among low-income households.
